# Dengue Virus Non-Structural Protein 5 as a Versatile, Multi-Functional Effector in Host–Pathogen Interactions

**DOI:** 10.3389/fcimb.2021.574067

**Published:** 2021-03-18

**Authors:** Priya Bhatnagar, Gopinathan Pillai Sreekanth, Kaja Murali-Krishna, Anmol Chandele, Ramakrishnan Sitaraman

**Affiliations:** ^1^ Department of Biotechnology, TERI School of Advanced Studies, New Delhi, India; ^2^ ICGEB-Emory Vaccine Centre, International Centre for Genetic Engineering and Biotechnology (ICGEB), New Delhi, India; ^3^ Department of Paediatrics and Emory Vaccine Centre, Emory University School of Medicine, Atlanta, GA, United States

**Keywords:** Flavivirus, NS5, moonlighting proteins, signaling pathways, protein–protein interactions (PPIs), antiviral immunity, apoptosis, spliceosome

## Abstract

Dengue is emerging as one of the most prevalent mosquito-borne viral diseases of humans. The 11kb RNA genome of the dengue virus encodes three structural proteins (envelope, pre-membrane, capsid) and seven non-structural proteins (NS1, NS2A, NS2B, NS3, NS4A, NS4B, and NS5), all of which are translated as a single polyprotein that is subsequently cleaved by viral and host cellular proteases at specific sites. Non-structural protein 5 (NS5) is the largest of the non-structural proteins, functioning as both an RNA-dependent RNA polymerase (RdRp) that replicates the viral RNA and an RNA methyltransferase enzyme (MTase) that protects the viral genome by RNA capping, facilitating polyprotein translation. Within the human host, NS5 interacts with several proteins such as those in the JAK-STAT pathway, thereby interfering with anti-viral interferon signalling. This mini-review presents annotated, consolidated lists of known and potential NS5 interactors in the human host as determined by experimental and computational approaches respectively. The most significant protein interactors and the biological pathways they participate in are also highlighted and their implications discussed, along with the specific serotype of dengue virus as appropriate. This information can potentially stimulate and inform further research efforts towards providing an integrative understanding of the mechanisms by which NS5 manipulates the human-virus interface in general and the innate and adaptive immune responses in particular.

## Introduction

Dengue is a global epidemic resulting in over 100 million clinical cases globally each year with symptoms ranging from fever to hemorrhage and/or shock that can be fatal, especially among children (Guzman et al., 2010; Bhatt et al., 2013). The disease is caused by four distinct dengue virus (DENV) serotypes (DENV-1, 2, 3, 4). DENV is a positive-strand RNA virus that belongs to the genus Flavivirus, family Flaviviridae. The genome encodes three structural (Env, PreM, Capsid) and seven non-structural (NS1, NS2A, NS2B, NS3, NS4A, NS4B, and NS5) proteins. Of these NS1 interacts with NS4A/B and promotes viral replication (Chen et al., 2018; Płaszczyca et al., 2019), NS3 performs helicase and protease functions (Swarbrick et al., 2017), NS4A induces autophagy (McLean et al., 2011), and NS4B facilitates dissociation of NS3 helicase from viral RNA (Umareddy et al., 2006).

NS5 is the largest and the most conserved DENV protein. It serves two important functions: one is the RNA-dependent RNA polymerase (RdRp) activity that is required for viral replication (Iglesias et al., 2011). The second is RNA methyltransferase (MTase) activity important for RNA capping during polyprotein translation (Liu et al., 2010; Klema et al., 2016). Additionally, NS5 forms an RNA replicase complex with NS3 in the endoplasmic reticulum during viral replication. After replication, NS5 dissociates from NS3 and translocates to the nucleus (Kapoor et al., 1995). So far, nuclear translocation has been reported for DENV-2 and -3 serotypes (Brooks et al., 2002; Hannemann et al., 2013). Yeast two-hybrid (Y2H) studies suggest that nuclear translocation may occur because the nuclear import receptor importin-β competes with DENV-NS3 for binding with NS5 (Johansson et al., 2001). While the nuclear accumulation of NS5 does not seem to be essential for viral replication (Kumar et al., 2013), it appears to be linked to an increase in the production of the cytokine IL-8 that has been historically correlated with severe dengue (Medin et al., 2005).

Given that NS5 is important for viral replication and serves as a major target for cytotoxic T cell responses (Duangchinda et al., 2010; Alves et al., 2016), there has been much interest to target it for vaccine development and anti-viral interventions. Mutational studies on the NS5-MTase domain identified several residues that are likely to be critical in viral replication (Kroschewski et al., 2008). 2′-O-methylation of the viral RNA is crucial for the dampening of host immune responses at the early stages of the viral life cycle. Abrogation of the 2′-O-MTase by changing a single amino-acid (E216A) results in an earlier activation of anti-viral responses exemplified by RIG-I (a sensor of foreign RNA), IL-8 (a pro-inflammatory cytokine), and IFIT2 (an interferon-induced protein that inhibits translation) leading to viral attenuation (Chang et al., 2016). Several inhibitors of MTase and RdRp activities have been identified by large-scale *in vitro* screening (reviewed by (Lim et al., 2015)). Additionally, NS5 interacts with host proteins such as STAT2 that are critical for type 1 interferon (IFN-I) signaling and innate responses and inhibits host anti-viral responses (reviewed recently by (Ashour et al., 2009; El Sahili and Lescar, 2017). In addition to such well-studied instances, recent high-throughput studies in a variety of experimental systems, as well as bioinformatic analyses, suggest that NS5 interacts with a diverse spectrum of host proteins (Rawlinson et al., 2006; El Sahili and Lescar, 2017; Amemiya et al., 2019). The goal of this review is to provide the interested researcher with a consolidated, annotated list of known and potential NS5-interacting human proteins obtained from multiple studies, highlight significant candidate interactors and situate them in specific biological contexts wherever possible. Additionally, information on the serotype of the viral strain (DENV1–4) used in the cited studies have been retained and highlighted wherever appropriate.

## Compilation of Ns5-Interacting Host Proteins From the Literature

While some of the NS5 interacting host proteins such as STAT2 are well-known, and extensively reviewed (Ashour et al., 2009; El Sahili and Lescar, 2017) the goal of our efforts here is to compile a comprehensive list of NS5 interacting host proteins. We approached this by compilation of NS5 interacting human proteins a) discovered by experimental pull-down studies reported in the literature; b) curated in databases (bioinformatics and Y2H studies). We briefly elaborate on each of these approaches followed by a list of NS5 interacting proteins compiled through these approaches. Finally, we comment on the gaps in our understanding of the role of these interactions and directions that future research in the field could take.

### Pull-Down Studies

Typically, pull-down studies have used cell lines that are infected with defined DENV serotypes and/or strains or transfected by DENV-NS5 protein. While this approach has the advantage of direct evaluation of protein-protein interactions (PPIs) the result may be influenced by the cell line used, and the serotype/strain used for infection/transfection. One study infected HEK 293T and Huh7 cells with strep-tagged full-length DENV-2 (strain 16681) and determined 53 binding partners (De Maio et al., 2016). Another study transfected HEK 293T cells with NS5 of DENV-2 (strain 16681) followed by affinity purification-mass spectroscopy (AP-MS) (Shah et al., 2018), and the data so generated were analysed using MiST (mass spectrometry interaction statistics (Verschueren et al., 2015) and CompPASS (Comparison of Multiple Protein Alignments with Assessment of Statistical Significance (Sadreyev and Grishin, 2003). This resulted in the identification of 26 NS5-interacting host proteins. Another study by Carpp et al. (2014) identified 53 interactors of NS5 using HEK293-T cell line. As the addition of affinity tags to the coding sequences of NS5 and NS3 prevented the production of recombinant virions, they used the I-DIRT (isotopic differentiation of interactions as random or targeted) immunoaffinity purification method (Tackett et al., 2005). Cell lines grown in the normal medium were transfected with GFP-tagged NS3/NS5 followed by DENV-2 infection. Cell lines growing in media containing isotopically labeled arginine and lysine (^13^C_6_, ^15^N_4_) were mock-transfected followed by DENV2 infection. After lysis of both samples, equal amounts of the extracts were mixed. This approach distinguishes between pre-lysis and post-lysis interactions by identifying non-specific post-lysis interactions due to the increased proportion of heavy relative to light isotopes (Carpp et al., 2014). In a fourth pull-down study (Poyomtip et al., 2016), a full-length DENV-2 construct (strain 16681) with tandem affinity purification (TAP)-tagged NS5 containing a poly-histidine and FLAG tags (inserted following N173 in MTase domain of NS5) was propagated in BHK21 cells followed by infection in Huh-7 cells. The NS5 complexes were isolated *via* FLAG-IP and analyzed by mass spectroscopy. This study revealed 97 NS5 interactors, prominent among them being heterogeneous nuclear ribonucleoproteins (hnRNPs) and proteins involved in lipid metabolism (Poyomtip et al., 2016).

### Information From Databases and Yeast Two-Hybrid Studies

We used P-HIPSTer (pathogen-host interactome prediction using structure similarity; http://phipster.org) which is a database of computationally predicted PPIs compiled for a set of 1,001 fully sequenced human-infecting viruses. The predictions are based on protein structural similarity and homology modeling, exploiting both sequence and structure-based information to infer interactions between pathogen and human proteins (Lasso et al., 2019). This database employs the extensively validated Pre-PPI (predicting protein-protein interactions) algorithm for its predictions. Additionally, we also used DenvInt (https://denvint.000webhostapp.com/) which is a dengue-specific database of serotype-related experimental evidence of PPIs based entirely on experimental evidence (Dey and Mukhopadhyay, 2017). It curates data from Y2H, bacterial two-hybrid, pull-down, and co-localization experiments (Khadka et al., 2011; Le Breton et al., 2011; Mairiang et al., 2013). This database indicates that of all DENV proteins, NS5 interacts with the largest number of human proteins (152).

**Figure 1:**
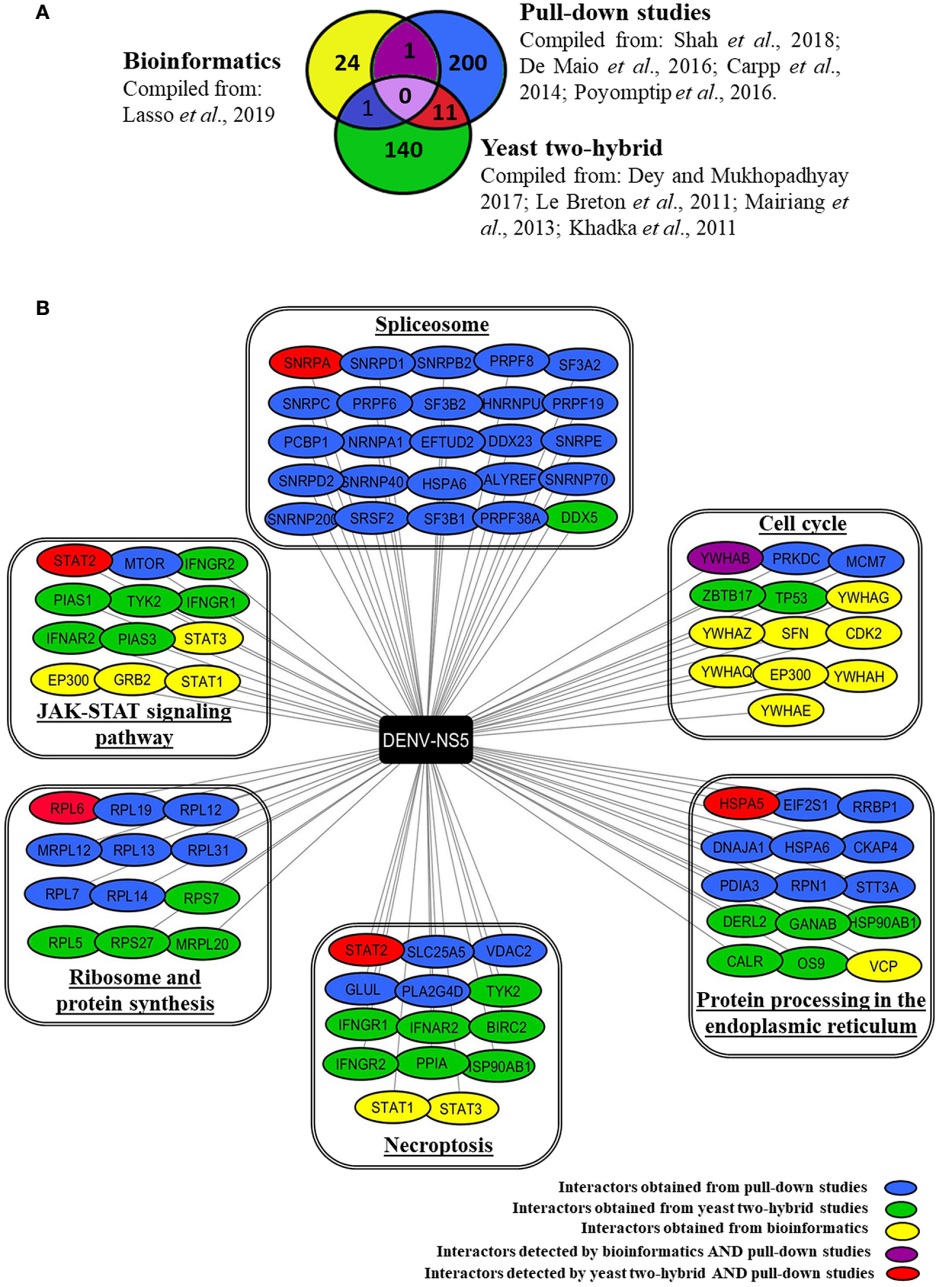
Human interacting partners of DENV-NS5 curated from various experimental studies and databases. **(A)** The Venn diagram indicates the number of DENV-NS5 interacting proteins that are shared with and/or unique to PPI studies in the literature viz., yeast-two-hybrid studies, pull-down studies, and bioinformatics. Yeast-two-hybrid data were curated from the DenvInt database, bioinformatics-based data was obtained from P-HIPSTer, and pull-down data has been derived from published data sources. All cited sources and extended data are compiled and listed in **Supplementary Table 1**. **(B)** Some of the NS5 interactors involvement in key KEGG pathways as obtained using the WEB-based GEne SeT AnaLysis Toolkit. The interactors are grouped in boxes based on the key pathways that they are involved in as obtained from KEGG. The proteins are color-coded according to the method used for their identification. A list of all significant pathways with a false discovery rate (FDR) < 0.05 is given in **Supplementary Table 2**. The complete results of the GO filtering are shown in **Supplementary Figure 1**. SPTAN1 is the only protein detected by both bioinformatics and yeast two-hybrid experiments but has not been shown here because it was associated with a false discovery rate > 0.05 which is the threshold for our compilation.

Based on databases and published studies, we have compiled a total of 377 proteins that are known/predicted to interact with DENV-NS5 protein. **Figure 1A** depicts the number of interacting proteins identified by each of the above-stated methods of discovery. **Supplementary Table 1** provides an extensive annotated list of these different NS5 interactors along with the serotype and method by which these are deduced. The minimal overlap of the NS5 interactors deduced by these different approaches may be due to the differences in overall methodologies. Pull-down studies use specific cell lines, viruses, or viral strains as explained above. Overexpression of target proteins in cell lines through transfection does not mimic the actual viral infection scenario, pull-down studies can lead to the precipitation of protein complexes, whose components may not all directly interact with the target protein. Extensive washing steps involved in this protocol may lead to dissociation of weak or transitory interactors. Yeast two-hybrid, though a rapid technique for large scale screening of PPIs, does not truly reflect the sub-cellular localization of the expressed protein or the abundance of the interacting proteins inside the cell. However, though bioinformatics analysis has the advantage of taking into consideration many viral variants and the conserved amino acids among them, which is usually not feasible in experimental systems that rely on a limited set of viral strains, it can produce potentially false-positive results.

Using the available data from the combination of approaches described above, we determined the biological pathways that these interactors are potentially involved with using the KEGG database, Gene Ontology (GO) analysis, and WEB-based GEne SeT AnaLysis Toolkit (Liao et al., 2019) available at http://www.webgestalt.org/. **Supplementary Figure 1** provides top biological processes, cellular components, and functions of these NS5 interactors. **Supplementary Table 2** provides an extensive list of the top pathways with a false discovery rate < 0.05. **Figure 1B** outlines some of the major pathways that are enriched for NS5 interacting host proteins pertaining to JAK-STAT signaling pathway, spliceosome, cell cycle, protein processing in ER, necroptosis, and protein synthesis. These are further elaborated in the section below.

## Critical Comments on The Growing List of Ns5-Interacting Human Proteins

The most studied NS5 interactor is STAT2. Su et al. reported that SUMOylation of DENV-NS5 is vital for suppressing STAT2-mediated IFN responses (Su et al., 2016). Excellent reviews are available on this subject and thus we are not elaborating on this aspect further (El Sahili and Lescar, 2017). Interestingly, the expanding list of NS5 interactors started revealing several other proteins that are involved in JAK-STAT signaling as outlined in **Figure 1B**, some of which are deduced by pull-down studies (STAT2, MTOR), some by Y2H studies (PIAS1, PIAS3, IFNAR2, TYK2 IFNGR1, IFNGR2), and the others by bioinformatics approaches (STAT1, STAT3, GRB2, EP300) (See **Figure 1B**). It is interesting to note that dengue NS5 not only interacts with IFN-α/β Receptor Subunit 2 (IFNAR2) but also interacts with interferon-gamma receptors 1 and 2 (IFNGR1, IFNGR2). This raises the possibility that NS5, in addition to interfering with the JAK-STAT signaling pathway (Best, 2017), may also interfere with the action of Type I IFN’s or IFNγ, which are the key innate and adaptive anti-viral cytokine respectively. Notably, a case-control study that sequenced the DENV-1 NS5 gene in 31 patients of varying disease severity found that polymorphisms corresponding to amino-acids 124 and 166 (I124M and G166S respectively) correlated with increased disease severity in what was designated as viral “clade 2” relative to “clade1” by the researchers. Computational analysis of these amino acid variants indicated that this effect was probably due to the stronger interaction of clade 2 NS5 with the type-I interferon receptor and Janus kinase-1 (JAK-1), eventually suppressing JAK-STAT signaling (Delgado-Enciso et al., 2018) thereby dampening key pathways of the innate immune response. Further studies are needed to understand which domain of NS5 interacts with these different proteins, and what the direct and indirect effects of these interactions are.

It is interesting to note that the list of NS5 interactors constitutes a large number of proteins involved in the spliceosome machinery. Pre-mRNA splicing is a critical mechanism of gene regulation in eukaryotic cells since a majority of protein-encoding transcripts are alternatively spliced (Lee and Rio, 2015). As mRNA splicing is altered in various pathological conditions, it is a potential target for therapeutic intervention using small molecules (Effenberger et al., 2017). De Maio et al. (2016) showed NS5 binds to spliceosome complexes and reduces the efficiency of pre-mRNA processing. Using proteomic analysis and functional experiments, this study demonstrated that NS5 interacts with CD2BP2 and DDX23 from the U5 small nuclear ribonucleoprotein (snRNP) particle to modify the inclusion/exclusion ratio of alternative splicing events, altering the mRNA isoform abundance of known antiviral factors such as CFTR, EDI, and Bclx (De Maio et al., 2016). DENV-NS5 targets nuclear RNA-binding protein 10 (RBM10) for proteasomal degradation. RBM10 regulates alternative splicing, favoring anti-viral mRNA isoforms of proteins such as spermidine/spermine-N1-acetyltransferase (SAT1) (Pozzi et al., 2020). Its degradation favors pro-viral isoforms, aiding viral replication; however, it is unknown whether this is a direct interaction or not. Interestingly, in this regard, it is interesting to note that NS5 reduces the splicing efficiency of endogenous RIG-I mRNA, and also increases the expression of dominant-negative forms of IKKϵ during DENV infection, all leading to maintenance of the pro-viral conditions in the cell (De Maio et al., 2016). The NS5 protein of ZIKA and JEV has also been shown to interact with spliceosome-associated proteins (Kovanich et al., 2019). Considering these, it is proposed that NS5 interaction with the spliceosome machinery could be an immune suppression strategy (De Maio et al., 2016). Some recent studies have shed new light on other NS5-interacting human proteins. For example, a ChIP assay study of DENV-2 NS5-transfected HEK293 cells found increased binding of NF-κB on the RANTES promoter than in cells mock-transfected with the empty vector (Khunchai et al., 2015). Elevated RANTES expression in NS5 transfected HEK-293 was validated at both mRNA and protein levels using real-time PCR and ELISA respectively (Khunchai et al., 2015).

NS5 interacts with a host protein, death domain associated protein 6 (Daxx) competitively, which dissociates the Daxx-NF-kB complex. This leads to an increased availability of NF-kB to bind with RANTES promotor and increases RANTES expression (Khunchai et al., 2012). This is very interesting given the observation that NS5 upregulates RANTES which is a key cytokine produced in severe dengue cases (Khunchai et al., 2015; Soo et al., 2017). However, a different study showed that NS5 transfection of HEK293 cells led to upregulation of IL-8 *via* activation of CAAT/enhancer-binding protein (c/EBP) (Medin et al., 2005). Further studies are needed to understand how NS5 transfection influences NF-κB given that NF-κB is a pleiotropic factor that can affect multiple biological processes such as cytokine production, transcription, translation, and apoptosis. In this regard, it is interesting to note that many of the apoptosis-related proteins (e.g., BIRC2; SPTAN1; TUBAL3, etc.) are also shown to interact with dengue NS5 (**Supplementary Table 1**).

Interestingly, NS5 interacts with several proteins that are typically associated with lipid metabolism (fatty acid synthase, hydroxysteroid (17β) dehydrogenase 12, pyruvate carboxylase, ATP citrate lyase). This indicates that NS5 may have a direct role in influencing lipid metabolism (Heaton and Randall, 2010; Carpp et al., 2014; Poyomtip et al., 2016). Further understanding of the role of NS5 in these pathways is important given that lipid metabolism is necessary for viral replication (Melo et al., 2018).

Some of the recent emerging studies are beginning to indicate that NS5 has a causal link in autophagy *via* influencing a host deubiquitinase protein, USP42 expression *via* increased microRNA, miR-590 (Mishra et al., 2019) and TRAF-6 (Pu et al., 2017). However, the interacting partners of NS5 involved in these processes are yet to be identified.

An interesting line of studies in the recent past suggests that NS5 also interacts with promyelocytic leukemia-nuclear bodies **(**PML-NBs) that are typically involved in several cellular processes including antiviral response (Lallemand-Breitenbach and de Thé, 2010; Khunchai et al., 2012; Giovannoni et al., 2015).

These various lines of evidence indicate that besides the well-known dampening of the initial anti-viral response, NS5 can interact with several other host proteins to influence other aspects of host cell physiology as well. The precise effect of these NS5-host protein interactions on the overall survival and propagation of the virus as well as on the host innate and adaptive immune responses remains to be determined.

## Future Prospects

DENV-NS5 interactors participate in a variety of biological processes, most importantly JAK-STAT signalling, RNA processing, cell cycle progression, necroptosis, protein synthesis, and protein processing in the ER among others. DENV-NS5 is an attractive target for drugs and small molecules to inhibit viral replication (Rawlinson et al., 2006; Lim et al., 2015; Shimizu et al., 2019; Troost and Smit, 2020). RNA interference (RNAi)-based approaches have been explored for therapeutic potential against a variety of viral infections, including dengue [reviewed in (Stein and Shi, 2008; Arbuthnot, 2010; Levanova and Poranen, 2018)]. Validating the top hits among the listed NS5-interactors by RNAi in human cell lines and observing the effect of such inhibition of specific host proteins on viral viability or pathogenesis could rapidly identify promising host proteins for disease management. Stepwise investigation of the utility of knocking down interactor-protein levels *via* RNAi and/or deploying interactor decoys to hamper the NS5-interaction with specific host proteins suggest themselves as potential avenues for further clinical research. Some of the NS5-interactors that modulate immune functions or lipid metabolism may serve as potential targets (Canard, 2011). The choice of host protein(s) would be critical, and those involved in more specialized pathways like necroptosis or cytokine production may be preferred over those involved in essential processes like protein synthesis or RNA processing to minimize collateral damage to the host. In case of dengue, RNAi approaches have obtained promising results by targeting TNF-α in cell culture and mice (Subramanya et al., 2010). Furthermore, cell line-based RNAi studies targeting Hsp60 (Padwad et al., 2009), proteins involved in membrane trafficking (Ang et al., 2010) and protein processing in the ER (Savidis et al., 2016), and the IFN-λ receptor 1 (Hsu et al., 2016) indicate that an appropriate choice of host protein, can favorably influence the course of viral infection and disease pathogenesis. Since most of the experimental data on NS5-interacting host proteins available to date are for DENV-2, it would help to learn about serotype-specific differences to fine-tune drug usage. Further investigation of NS5-host protein interactions and their outcomes vis-à-vis viral infection and disease pathogenesis can potentially open novel avenues for effective viral therapy and/or clinical management.

## Author Contributions

KM-K, AC, and RS contributed to the conception and design of the review. PB organized the database and performed the analysis. PB and GS wrote the first draft of the manuscript. All authors contributed to the article and approved the submitted version.

## Funding

PB was a recipient of a CSIR-UGC doctoral fellowship from the National Human Resource Development for S&T, Government of India [Sr. No. 2121330697; Ref. No. 22/12/2013(ii)EU-V]. Research in the laboratories of KM-K and AC is supported by the NIH-DBT, Human Immunophenotyping Project Consortium (HIPC) grant (BT/PR30260/MED/15/194/2018), and the DBT-Biotechnology Industry Research Assistance Council (BIRAC) grant (BT/NBM0099/02/18).

## Conflict of Interest

The authors declare that the research was conducted in the absence of any commercial or financial relationships that could be construed as a potential conflict of interest.

## References

[B1] Alves R. P. D. S. Pereira L. R. Fabris D. L. N. Salvador F. S. Santos R. A. Zanotto P. M. D. A.. (2016). Production of a Recombinant Dengue Virus 2 NS5 Protein and Potential Use as a Vaccine Antigen. Clin. Vaccine Immunol. 23, 460–469. 10.1128/CVI.00081-16 27030586 PMC4895003

[B2] Amemiya T. Gromiha M. M. Horimoto K. Fukui K. (2019). Drug repositioning for dengue haemorrhagic fever by integrating multiple omics analyses. Sci. Rep. 9, 523. 10.1038/s41598-018-36636-1 30679503 PMC6346040

[B3] Ang F. Wong A. P. Ng M. M. Chu J. J. (2010). Small interference RNA profiling reveals the essential role of human membrane trafficking genes in mediating the infectious entry of dengue virus. Virol. J. 7, 24. 10.1186/1743-422X-7-24 20122152 PMC2825209

[B4] Arbuthnot P. (2010). Harnessing RNA interference for the treatment of viral infections. Drug News Perspect. 23, 341–350. 10.1358/dnp.2010.23.6.1437713 20697601

[B5] Ashour J. Laurent-Rolle M. Shi P. Y. Garcia-Sastre A. (2009). NS5 of dengue virus mediates STAT2 binding and degradation. J. Virol. 83, 5408–5418. 10.1128/JVI.02188-08 19279106 PMC2681973

[B6] Best S. M. (2017). The Many Faces of the Flavivirus NS5 Protein in Antagonism of Type I Interferon Signaling. J. Virol. 91(3):e01970-16. 10.1128/JVI.01970-16 27881649 PMC5244349

[B7] Bhatt S. Gething P. W. Brady O. J. Messina J. P. Farlow A. W. Moyes C. L.. (2013). The global distribution and burden of dengue. Nature 496, 504–507. 10.1038/nature12060 23563266 PMC3651993

[B8] Brooks A. J. Johansson M. John A. V. Xu Y. Jans D. A. Vasudevan S. G. (2002). The interdomain region of dengue NS5 protein that binds to the viral helicase NS3 contains independently functional importin beta 1 and importin alpha/beta-recognized nuclear localization signals. J. Biol. Chem. 277, 36399–36407. 10.1074/jbc.M204977200 12105224

[B9] Canard B. (2011). Antiviral Research and Development Against Dengue Virus. WHO Rep. 1–101. Available at https://www.who.int/tdr/research/ntd/dengue/dengue_full_length_report.pdf

[B10] Carpp L. N. Rogers R. S. Moritz R. L. Aitchison J. D. (2014). Quantitative proteomic analysis of host-virus interactions reveals a role for Golgi brefeldin A resistance factor 1 (GBF1) in dengue infection. Mol. Cell Proteomics 13, 2836–2854. 10.1074/mcp.M114.038984 24855065 PMC4223476

[B11] Chang D. C. Hoang L. T. Naim A. N. M. Dong H. Schreiber M. J. Hibberd M. L.. (2016). Evasion of early innate immune response by 2′-O-methylation of dengue genomic RNA. Virology 499, 259–266. 10.1016/j.virol.2016.09.022 27716465 PMC7172056

[B12] Chen H.-R. Lai Y.-C. Yeh T.-M. (2018). Dengue virus non-structural protein 1: a pathogenic factor, therapeutic target, and vaccine candidate. J. Biomed. Sci. 25, 58. 10.1186/s12929-018-0462-0 30037331 PMC6057007

[B13] De Maio F. A. Risso G. Iglesias N. G. Shah P. Pozzi B. Gebhard L. G.. (2016). The Dengue Virus NS5 Protein Intrudes in the Cellular Spliceosome and Modulates Splicing. PLoS Pathog. 12, e1005841. 10.1371/journal.ppat.1005841 27575636 PMC5004807

[B14] Delgado-Enciso I. Lopez-Lemus U. A. Valcarcel-Gamino J. A. Rodriguez-Sanchez I. P. Valle-Reyes S. Martinez-Fierro M. L.. (2018). Dengue virus-1 NS5 genetic variant associated with a severe clinical infection: Possible reduction of the innate immune response by inhibition of interferon type 1 and the Janus kinase-signal transducer and activator of transcription signaling pathway. Int. J. Mol. Med. 41, 2263–2269. 10.3892/ijmm.2018.3395 29344662

[B15] Dey L. Mukhopadhyay A. (2017). DenvInt: A database of protein–protein interactions between dengue virus and its hosts. PLoS Negl. Trop. Dis. 11, e0005879. 10.1371/journal.pntd.0005879 29049286 PMC5648114

[B16] Duangchinda T. Dejnirattisai W. Vasanawathana S. Limpitikul W. Tangthawornchaikul N. Malasit P.. (2010). Immunodominant T-cell responses to dengue virus NS3 are associated with DHF. Proc. Natl. Acad. Sci. U. S. A. 107, 16922–16927. 10.1073/pnas.1010867107 20837518 PMC2947904

[B17] Effenberger K. A. Urabe V. K. Jurica M. S. (2017). Modulating splicing with small molecular inhibitors of the spliceosome. Wiley Interdiscip. Rev. RNA 8, e1381. 10.1002/wrna.1381 PMC5253128 27440103

[B18] El Sahili A. Lescar J. (2017). Dengue Virus Non-Structural Protein 5. Viruses 9(4):91. 10.3390/v9040091 PMC5408697 28441781

[B19] Giovannoni F. Damonte E. B. Garcia C. C. (2015). Cellular promyelocytic leukemia protein is an important dengue virus restriction factor. PLoS One 10 (5), e0125690. 10.1371/journal.pone.0125690. 25962098 PMC4427460

[B20] Guzman M. G. Halstead S. B. Artsob H. Buchy P. Farrar J. Gubler D. J.. (2010). Dengue: a continuing global threat. Nat. Rev. Microbiol. 8, S7–S16. 10.1038/nrmicro2460 21079655 PMC4333201

[B21] Hannemann H. Sung P.-Y. Chiu H.-C. Yousuf A. Bird J. Lim S. P.. (2013). Serotype-specific differences in dengue virus non-structural protein 5 nuclear localization. J. Biol. Chem. 288, 22621–22635. 10.1074/jbc.M113.481382 23770669 PMC3829348

[B22] Heaton N. S. Randall G. (2010). Dengue virus-induced autophagy regulates lipid metabolism. Cell Host Microbe 8, 422–432. 10.1016/j.chom.2010.10.006 21075353 PMC3026642

[B23] Hsu Y. L. Wang M. Y. Ho L. J. Lai J. H. (2016). Dengue virus infection induces interferon-lambda1 to facilitate cell migration. Sci. Rep. 6, 24530. 10.1038/srep24530 27456172 PMC4960520

[B24] Iglesias N. G. Filomatori C. V. Gamarnik A. V. (2011). The F1 motif of dengue virus polymerase NS5 is involved in promoter-dependent RNA synthesis. J. Virol. 85, 5745–5756. 10.1128/JVI.02343-10 21471248 PMC3126321

[B25] Johansson M. Brooks A. J. Jans D. A. Vasudevan S. G. (2001). A small region of the dengue virus-encoded RNA-dependent RNA polymerase, NS5, confers interaction with both the nuclear transport receptor importin-β and the viral helicase, NS3. J. Gen. Virol. 82, 735–745. 10.1099/0022-1317-82-4-735 11257177

[B26] Kapoor M. Zhang L. Ramachandra M. Kusukawa J. Ebner K. E. Padmanabhan R. (1995). Association between NS3 and NS5 proteins of dengue virus type 2 in the putative RNA replicase is linked to differential phosphorylation of NS5. J. Biol. Chem. 270, 19100–19106. 10.1074/jbc.270.32.19100 7642575

[B27] Khadka S. Vangeloff A. D. Zhang C. Siddavatam P. Heaton N. S. Wang L.. (2011). A physical interaction network of dengue virus and human proteins. Mol. Cell Proteomics 10, M111.012187. 10.1074/mcp.M111.012187 PMC3237087 21911577

[B28] Khunchai S. Junking M. Suttitheptumrong A. Yasamut U. Sawasdee N. Netsawang J.. (2012). Interaction of dengue virus nonstructural protein 5 with Daxx modulates RANTES production. Biochem. Biophys. Res. Commun. 423, 398–403. 10.1016/j.bbrc.2012.05.137 22664104

[B29] Khunchai S. Junking M. Suttitheptumrong A. Kooptiwut S. Haegeman G. Limjindaporn T.. (2015). NF-κB is required for dengue virus NS5-induced RANTES expression. Virus Res. 197, 92–100. 10.1016/j.virusres.2014.12.007 25523420

[B30] Klema V. J. Ye M. Hindupur A. Teramoto T. Gottipati K. Padmanabhan R.. (2016). Dengue Virus Nonstructural Protein 5 (NS5) Assembles into a Dimer with a Unique Methyltransferase and Polymerase Interface. PLoS Pathog. 12, e1005451–e1005451. 10.1371/journal.ppat.1005451 26895240 PMC4760774

[B31] Kovanich D. Saisawang C. Sittipaisankul P. Ramphan S. Kalpongnukul N. Somparn P.. (2019). Analysis of the Zika and Japanese Encephalitis Virus NS5 Interactomes. J. Proteome Res. 18, 3203–3218. 10.1021/acs.jproteome.9b00318 31199156

[B32] Kroschewski H. Lim S. P. Butcher R. E. Yap T. L. Lescar J. Wright P. J.. (2008). Mutagenesis of the dengue virus type 2 NS5 methyltransferase domain. J. Biol. Chem. 283, 19410–19421. 10.1074/jbc.M800613200 18469001

[B33] Kumar A. Bühler S. Selisko B. Davidson A. Mulder K. Canard B.. (2013). Nuclear localization of dengue virus nonstructural protein 5 does not strictly correlate with efficient viral RNA replication and inhibition of type I interferon signaling. J. Virol. 87, 4545–4557. 10.1128/JVI.03083-12 23408610 PMC3624364

[B34] Lallemand-Breitenbach V. de Thé H. (2010). PML Nuclear Bodies. Cold Spring Harb. Perspect. Biol. 2, a000661. 10.1101/cshperspect.a000661 20452955 PMC2857171

[B35] Lasso G. Mayer S. V. Winkelmann E. R. Chu T. Elliot O. Patino-Galindo J. A.. (2019). A structure-informed atlas of human-virus interactions. Cell 178, 1526–1541.e1516. 10.1016/j.cell.2019.08.005 31474372 PMC6736651

[B36] Le Breton M. Meyniel-Schicklin L. Deloire A. Coutard B. Canard B. De Lamballerie X.. (2011). Flavivirus NS3 and NS5 proteins interaction network: a high-throughput yeast two-hybrid screen. BMC Microbiol. 11, 234. 10.1186/1471-2180-11-234 22014111 PMC3215679

[B37] Lee Y. Rio D. C. (2015). Mechanisms and Regulation of Alternative Pre-mRNA Splicing. Annu. Rev. Biochem. 84, 291–323. 10.1146/annurev-biochem-060614-034316 25784052 PMC4526142

[B38] Levanova A. Poranen M. M. (2018). RNA Interference as a Prospective Tool for the Control of Human Viral Infections. Front. Microbiol. 9:2151. 10.3389/fmicb.2018.02151 30254624 PMC6141738

[B39] Liao Y. Wang J. Jaehnig E. J. Shi Z. Zhang B. (2019). WebGestalt 2019: gene set analysis toolkit with revamped UIs and APIs. Nucleic Acids Res. 47, W199–W205. 10.1093/nar/gkz401 31114916 PMC6602449

[B40] Lim S. P. Noble C. G. Shi P. Y. (2015). The dengue virus NS5 protein as a target for drug discovery. Antiviral Res. 119, 57–67. 10.1016/j.antiviral.2015.04.010 25912817

[B41] Liu L. Dong H. Chen H. Zhang J. Ling H. Li Z.. (2010). Flavivirus RNA cap methyltransferase: structure, function, and inhibition. Front. Biol. (Beijing) 5, 286–303. 10.1007/s11515-010-0660-y 21927615 PMC3172701

[B42] Mairiang D. Zhang H. Sodja A. Murali T. Suriyaphol P. Malasit P.. (2013). Identification of new protein interactions between dengue fever virus and its hosts, human and mosquito. PLoS One 8, e53535–e53535. 10.1371/journal.pone.0053535 23326450 PMC3543448

[B43] McLean J. E. Wudzinska A. Datan E. Quaglino D. Zakeri Z. (2011). Flavivirus NS4A-induced autophagy protects cells against death and enhances virus replication. J. Biol. Chem. 286, 22147–22159. 10.1074/jbc.M110.192500 21511946 PMC3121359

[B44] Medin C. L. Fitzgerald K. A. Rothman A. L. (2005). Dengue virus nonstructural protein NS5 induces interleukin-8 transcription and secretion. J. Virol. 79, 11053–11061. 10.1128/JVI.79.17.11053-11061.2005 16103156 PMC1193580

[B45] Melo C. Delafiori J. Dabaja M. Z. De Oliveira D. N. Guerreiro T. M. Colombo T. E.. (2018). The role of lipids in the inception, maintenance and complications of dengue virus infection. Sci. Rep. 8, 11826. 10.1038/s41598-018-30385-x 30087415 PMC6081433

[B46] Mishra R. Sood V. Banerjea A. C. (2019). Dengue NS5 modulates expression of miR-590 to regulate ubiquitin-specific peptidase 42 in human microglia. FASEB Bioadv. 1, 265–278. 10.1096/fba.2018-00047 32123831 PMC6996368

[B47] Padwad Y. S. Mishra K. P. Jain M. Chanda S. Karan D. Ganju L. (2009). RNA interference mediated silencing of Hsp60 gene in human monocytic myeloma cell line U937 revealed decreased dengue virus multiplication. Immunobiology 214, 422–429. 10.1016/j.imbio.2008.11.010 19261350

[B48] Płaszczyca A. Scaturro P. Neufeldt C. J. Cortese M. Cerikan B. Ferla S.. (2019). A novel interaction between dengue virus nonstructural protein 1 and the NS4A-2K-4B precursor is required for viral RNA replication but not for formation of the membranous replication organelle. PLoS Pathog. 15, e1007736. 10.1371/journal.ppat.1007736 31071189 PMC6508626

[B49] Pozzi B. Bragado L. Mammi P. Torti M. F. Gaioli N, Gebhard L. G.. (2020). Dengue virus targets RBM10 deregulating host cell splicing and innate immune response. Nucleic Acids Res. 48, 6824–6838. 10.1093/nar/gkaa340 32432721 10.1093/nar/gkaa340 PMC7337517

[B50] Poyomtip T. Hodge K. Matangkasombut P. Sakuntabhai A. Pisitkun T. Jirawatnotai S.. (2016). Development of viable TAP-tagged dengue virus for investigation of host-virus interactions in viral replication. J. Gen. Virol. 97, 646–658. 10.1099/jgv.0.000371 26669909

[B51] Pu J. Wu S. Xie H. Li Y. Yang Z. Wu X.. (2017). miR-146a Inhibits dengue-virus-induced autophagy by targeting TRAF6. Arch. Virol. 162, 3645–3659. 10.1007/s00705-017-3516-9 28825144 PMC7086938

[B52] Rawlinson S. M. Pryor M. J. Wright P. J. Jans D. A. (2006). Dengue virus RNA polymerase NS5: a potential therapeutic target? Curr. Drug Targets 7, 1623–1638. 10.2174/138945006779025383 17168837

[B53] Sadreyev R. Grishin N. (2003). COMPASS: a tool for comparison of multiple protein alignments with assessment of statistical significance. J. Mol. Biol. 326, 317–336. 10.1016/S0022-2836(02)01371-2 12547212

[B54] Savidis G. Mcdougall W. M. Meraner P. Perreira J. M. Portmann J. M. Trincucci G.. (2016). Identification of Zika Virus and Dengue Virus Dependency Factors using Functional Genomics. Cell Rep. 16, 232–246. 10.1016/j.celrep.2016.06.028 27342126

[B55] Shah P. S. Link N. Jang G. M. Sharp P. P. Zhu T. Swaney D. L.. (2018). Comparative flavivirus-host protein interaction mapping reveals mechanisms of dengue and Zika virus pathogenesis. Cell 175, 1931–1945.e1918. 10.1016/j.cell.2018.11.028 30550790 PMC6474419

[B56] Shimizu H. Saito A. Mikuni J. Nakayama E. E. Koyama H. Honma T.. (2019). Discovery of a small molecule inhibitor targeting dengue virus NS5 RNA-dependent RNA polymerase. PLoS Negl. Trop. Dis. 13, e0007894. 10.1371/journal.pntd.0007894 31738758 PMC6886872

[B57] Soo K. M. Khalid B. Ching S. M. Tham C. L. Basir R. Chee H. Y. (2017). Meta-analysis of biomarkers for severe dengue infections. PeerJ 5, e3589. 10.7717/peerj.3589 28929009 PMC5602679

[B58] Stein D. A. Shi P. Y. (2008). Nucleic acid-based inhibition of flavivirus infections. Front. Biosci. 13, 1385–1395. 10.2741/2769 17981637

[B59] Su C. I. Tseng C. H. Yu C. Y. Lai M. M. C. (2016). SUMO Modification Stabilizes Dengue Virus Nonstructural Protein 5 To Support Virus Replication. J. Virol. 90, 4308–4319. 10.1128/JVI.00223-16 26889037 PMC4836324

[B60] Subramanya S. Kim S. S. Abraham S. Yao J. Kumar M. Kumar P.. (2010). Targeted delivery of small interfering RNA to human dendritic cells to suppress dengue virus infection and associated proinflammatory cytokine production. J. Virol. 84, 2490–2501. 10.1128/JVI.02105-08 20015996 PMC2820933

[B61] Swarbrick C. M. D. Basavannacharya C. Chan K. W. K. Chan S.-A. Singh D. Wei N.. (2017). NS3 helicase from dengue virus specifically recognizes viral RNA sequence to ensure optimal replication. Nucleic Acids Res. 45, 12904–12920. 10.1093/nar/gkx1127 29165589 PMC5728396

[B62] Tackett A. J. Degrasse J. A. Sekedat M. D. Oeffinger M. Rout M. P. Chait B. T. (2005). I-DIRT, a general method for distinguishing between specific and nonspecific protein interactions. J. Proteome Res. 4, 1752–1756. 10.1021/pr050225e 16212429

[B63] Troost B. Smit J. M. (2020). Recent advances in antiviral drug development towards dengue virus. Curr. Opin. Virol. 43, 9–21. 10.1016/j.coviro.2020.07.009 32795907

[B64] Umareddy I. Chao A. Sampath A. Gu F. Vasudevan S. G. (2006). Dengue virus NS4B interacts with NS3 and dissociates it from single-stranded RNA. J. Gen. Virol. 87, 2605–2614. 10.1099/vir.0.81844-0 16894199

[B65] Verschueren E. Von Dollen J. Cimermancic P. Gulbahce N. Sali A. Krogan N. J. (2015). Scoring Large-Scale Affinity Purification Mass Spectrometry Datasets with MiST. Curr. Protoc. Bioinf. 49, 8.19. 10.1002/0471250953.bi0819s49 PMC4378866 25754993

